# Process evaluation of a cluster randomised controlled trial to improve bronchiolitis management – a PREDICT mixed-methods study

**DOI:** 10.1186/s12913-021-07279-2

**Published:** 2021-11-29

**Authors:** Libby Haskell, Emma J. Tavender, Sharon O’Brien, Catherine L. Wilson, Franz E. Babl, Meredith L. Borland, Rachel Schembri, Francesca Orsini, Elizabeth Cotterell, Nicolette Sheridan, Ed Oakley, Stuart R. Dalziel, For the Paediatric Research in Emergency Departments International Collaborative (PREDICT) network, Australasia

**Affiliations:** 1 Children’s Emergency Department, Starship Children’s Hospital, Private Bag, Auckland, 92019 New Zealand; 2 Department of Paediatrics: Child and Youth Health, University of Auckland, Auckland, New Zealand; 3 Clinical Sciences, Murdoch Children’s Research Institute, Parkville, Melbourne, VIC Australia; 4 Departments of Paediatrics and Critical Care, University of Melbourne, Melbourne, Australia; 5 Emergency Department, Perth Children’s Hospital, Perth, WA Australia; 6 Curtin University, Perth, WA Australia; 7 Clinical Sciences, Murdoch Children’s Research Institute, Parkville, VIC Australia; 8 Emergency Department, The Royal Children’s Hospital, Melbourne, Australia; 9 Divisions of Emergency Medicine and Paediatrics, School of Medicine, University of Western Australia, Perth, WA Australia; 10 Clinical Epidemiology and Biostatistics, Melbourne Children’s Trials, Centre, Murdoch Children’s Research Institute, Melbourne, VIC Australia; 11 Armidale Rural Referral Hospital, Armidale, NSW Australia; 12 School of Rural Medicine, University of New England, Armidale, NSW Australia; 13 College of Health, Massey University, Auckland, New Zealand; 14 Departments of Surgery and Paediatrics: Child and Youth Health, University of Auckland, Auckland, New Zealand

## Abstract

**Background:**

Bronchiolitis is the most common reason for hospitalisation in infants. All international bronchiolitis guidelines recommend supportive care, yet considerable variation in practice continues with infants receiving non-evidence based therapies. We developed six targeted, theory-informed interventions; clinical leads, stakeholder meeting, train-the-trainer, education delivery, other educational materials, and audit and feedback. A cluster randomised controlled trial (cRCT) found the interventions to be effective in reducing use of five non-evidence based therapies in infants with bronchiolitis. This process evaluation paper aims to determine whether the interventions were implemented as planned (fidelity), explore end-users’ perceptions of the interventions and evaluate cRCT outcome data with intervention fidelity data.

**Methods:**

A pre-specified mixed-methods process evaluation was conducted alongside the cRCT, guided by frameworks for process evaluation of cRCTs and complex interventions. Quantitative data on the fidelity, dose and reach of interventions were collected from the 13 intervention hospitals during the study and analysed using descriptive statistics. Qualitative data identifying perception and acceptability of interventions were collected from 42 intervention hospital clinical leads on study completion and analysed using thematic analysis.

**Results:**

The cRCT found targeted, theory-informed interventions improved bronchiolitis management by 14.1%. The process evaluation data found variability in how the intervention was delivered at the cluster and individual level. Total fidelity scores ranged from 55 to 98% across intervention hospitals (mean = 78%; SD = 13%). Fidelity scores were highest for use of clinical leads (mean = 98%; SD = 7%), and lowest for use of other educational materials (mean = 65%; SD = 19%) and audit and feedback (mean = 65%; SD = 20%). Clinical leads reflected positively about the interventions, with time constraints being the greatest barrier to their use.

**Conclusion:**

Our targeted, theory-informed interventions were delivered with moderate fidelity, and were well received by clinical leads. Despite clinical leads experiencing challenges of time constraints, the level of fidelity had a positive effect on successfully de-implementing non-evidence-based care in infants with bronchiolitis. These findings will inform widespread rollout of our bronchiolitis interventions, and guide future practice change in acute care settings.

**Trial registration:**

Australian and New Zealand Clinical Trials Registry: ACTRN12616001567415.

**Supplementary Information:**

The online version contains supplementary material available at 10.1186/s12913-021-07279-2.

## Background

Bronchiolitis is the most common respiratory condition affecting infants. It is the leading cause of admission into hospital in infants less than 1 year of age in developed countries [1]. Management is well defined [1] with international guidelines consistently recommending respiratory and hydration support [2–5]. Despite high quality evidence of no benefit and potential harm from the use of chest x-ray (CXR), salbutamol, antibiotics, glucocorticoids and adrenaline, these five therapies continue to be widely used. In Australia and New Zealand, data from over 3400 presentations to seven hospitals show that at least one in five therapies were used at least once in 27 to 48% of bronchiolitis admissions [6]. These data are consistent with comparisons in the United Kingdom, North America, and Europe [7] and highlight the gap between evidence and current clinical practice that exists internationally.

Implementation research is the scientific study of methods to promote the uptake of research into routine practice, including the development and evaluation of interventions designed to reduce the evidence-practice gap [8]. Dissemination of clinical practice guidelines alone is seldom sufficient to drive change in practice with more active and targeted strategies required for change to occur [9]. Using theories of behaviour change and addressing both the barriers and enhancers of recommended practice are more likely to be effective [10, 11]. De-implementation or reducing the use of low-value healthcare has received less attention than implementation and is often considered more difficult. Healthcare systems are urged to embrace learnings from implementation science initiatives to date, to avoid repeating previous efforts and supporting development of a de-implementation international network [12].

In response to the identified practice variation in the treatment of infants with bronchiolitis, and recognising the importance of reducing the use of low-value and inappropriate therapies [13], targeted theory-informed interventions were developed aiming to increase compliance with five key recommendations from the Australasian Bronchiolitis Guideline [5]. Interventions were developed using a stepped approach, addressing factors influencing bronchiolitis management previously identified during qualitative clinician interviews [14] utilising the Theoretical Domains Framework (TDF) [15]. This validated framework incorporates a wide range of behaviour change theories for use in implementation research with demonstrated track record in explanatory and predictive powers across healthcare settings [16]. Guidance has been developed to inform the choice of behaviour change techniques most likely to tackle identified issues [17, 18]. The six bronchiolitis interventions chosen and developed were: 1. Clinical leads; 2. Stakeholder meeting; 3. Train-the-trainer workshop; 4. Educational intervention delivery; 5. Additional educational and promotional materials; 6. Audit and feedback. Table 1 details the bronchiolitis interventions and causal assumptions. Interventions were evaluated in an international multi-centre cluster randomised controlled trial (cRCT) and demonstrated effectiveness at improving bronchiolitis management by 14.1% and de-implementing unnecessary and low-value management [19]. Our stepped design followed the recently described Choosing Wisely De-implementation Framework [12].

**Table 1:** Bronchiolitis intervention components

Intervention (timing of intervention)	Description and causal assumptions/rationale
Clinical leads (February 2017)	Four clinical leads, including one nursing and one medical lead in each of the emergency department and paediatric inpatient units for duration of study. Key tasks included attending train-the-trainer 1 day workshop, leading delivery of educational intervention and other educational materials to all staff, overseeing completion of monthly audit and delivery of feedback, and coordinating study requirements. Rationale: Provide consistent credible, influential, and trustworthy leadership; increase knowledge and skills through education, influence and persuasion; clinical leads ensured interdisciplinary and interdepartmental coverage.
Stakeholder meeting (February to March 2017)	Study team met with clinical leads to present Australasian Bronchiolitis Guideline, discuss international and local variation in bronchiolitis management, review local audit results, and discuss any anticipated local barriers, with the aim to gain buy-in. Rationale: Create hospital buy-in; provide feedback on current management; knowledge of own practice variation is likely to drive change; increase knowledge of intervention process; identify and address any potential barriers.
Train-the-trainer workshop (23 February 2017)	One-day workshop for clinical leads to discuss: Australasian Bronchiolitis Guideline and evidence underpinning recommendations, implementation, qualitative study identifying barriers and facilitators to bronchiolitis management, and development process of interventions. Demonstrated to clinical leads how to deliver educational intervention to their staff, outlined study data requirements and timeline, and facilitated planning time for clinical leads. Rationale: Improve knowledge; change beliefs; optimise professional interdisciplinary and interdepartmental relationships; motivate clinical leads as drivers of change.
Educational intervention delivery (1 May to 30 November 2017)	PowerPoint presentation designed with scripted messages addressing key findings from qualitative study using behaviour change techniques most likely to effect change. Education delivery overseen by clinical leads to nursing and medical staff using PowerPoint presentation. Aimed to train 80% of staff within first month and on-going education throughout duration of study ensuring all staff educated. Rationale: Improve knowledge; increase skills; change beliefs; feedback on performance; address barriers and enablers to evidence-based management; reinforce importance of evidence-based management and consequences of not following recommendations; positive reinforcement.
Use of other educational materials (1 May to 30 November 2017)	Clinician training video Rationale: Demonstrate/role model clinician behaviour; increase skill; provide motivation. Evidence fact sheets Rationale: Improve knowledge; change beliefs of clinicians. Promotional materials Rationale: Reminder/prompt of recommended management; feedback on performance; provide motivation. Parent/caregiver information Rationale: Improve knowledge; increase skill and confidence; provide encouragement and support.
Audit and feedback (1 May to 30 November 2017)	Monthly audits of the first 20 bronchiolitis presentations, with report produced showing individual hospital results compared with top-performing hospital. Report disseminated by clinical leads to their staff in verbal and written format; action planning with target setting encouraged. Rationale: Provide real-time feedback on targeted behaviours; motivate by benchmarking; promote goal/target specific action planning to optimise on-going improvement; increase knowledge; change beliefs.

A process evaluation was conducted alongside the cRCT. While RCTs are accepted as gold standard for evaluating intervention effectiveness [20], RCT results alone do not provide information on what worked, how, and why. Process evaluation of complex interventions (those having multiple active strategies) as in our cRCT, is required to open the “black box” on what may or may not have worked and why [20]. Evaluations undertaken alongside a trial can clarify the degree of implementation fidelity, how and why it worked (or didn’t work), and how interventions could be improved for subsequent programmes. Process evaluations are particularly important in multi-site trials where the same intervention is delivered yet received and utilised differently, and assists the interpretation of outcome results [21]. A systematic review of process evaluations found weak evidence-base in implementation studies, recommending using mixed-methods, theory-guided design, occurring both during and following implementation [22]. Integrating process and outcome analysis allows evaluations to explore possible associations between implementation strategies, delivery, receipt, and outcomes on effectiveness [23].

Process evaluation in acute paediatric settings, and in de-implementation are rare. However process evaluation is vital for improving broad dissemination of successful de-implementation interventions or identifying areas for improvement. This evaluation aims to provide insight in to the interventions and delivery, experiences of clinical leads, and how these findings relate to the bronchiolitis cRCT results. Specifically, the objectives being:

1. To evaluate the degree to which the bronchiolitis interventions were delivered as planned (fidelity, dose and reach).
2. To explore clinical lead perceptions of the interventions, execution of these in a real clinical setting and acceptability (participant perspective).
3. To explore relationships between intervention fidelity and effectiveness data from our cRCT results, drawing lessons for future de-implementation projects.

## Methods

### Study design

A pre-specified mixed-methods process evaluation was conducted alongside the Paediatric Research in Emergency Departments International Collaborative (PREDICT) bronchiolitis cRCT, the protocol and results are published elsewhere [19, 24]. This has been guided by a framework for process evaluation of cRCTs [23], the United Kingdom Medical Research Council (MRC) recommendations for complex interventions [20], and a systematic review of process evaluations in knowledge translation research [22]. Additional file 1: Appendix Table 1 details components and methods utilised in the process evaluation.

A quantitative component measured intervention fidelity, the degree to which the intervention was delivered as intended, dose and reach. A qualitative component examined clinical leads perceptions of the interventions via an online questionnaire. Both qualitative and quantitative findings are viewed together when interpreting findings.

### Study setting and participants

A total of 26 hospitals (clusters) across Australia and New Zealand were recruited [24]. Hospitals were randomised to intervention (*n* = 13) or control (*n* = 13). The emergency department (ED) and paediatric inpatient unit clinicians (nursing and medical) for each of the 13 intervention hospitals and clinical leads (nursing and medical in both departments) responsible for delivering the interventions were participants in the process evaluation. Intervention hospitals received targeted, theory-informed interventions. Control hospitals received an electronic and printed copy of the complete Australasian Bronchiolitis Guideline [5], representing usual practice for guideline dissemination at the time. Control hospitals received all interventions at the completion of the study. The implementation period was the Australian and New Zealand bronchiolitis season, 1st May 2017 to 30th November 2017.

### Bronchiolitis interventions

Intervention hospitals received interventions targeting nursing and medical clinicians who managed infants with bronchiolitis in the ED and paediatric inpatient units (Table 1). Interventions were developed using a stepped theory-informed approach: 1) five key evidence-based recommendations were identified from the Australasian Bronchiolitis Guideline [5], 2) a qualitative study of clinicians in Australia and New Zealand identified factors perceived to influence the treatment of infants with bronchiolitis [14] using the TDF [18], 3) findings from this study were mapped to behaviour change techniques most likely to effect change for the identified factors [17, 18], and 4) targeted interventions were developed to operationalise these behaviour change techniques for the ED and paediatric inpatient units by considering the feasibility, local relevance, and acceptability of the intervention components. Additional file 1: Appendix Table 2 details how the bronchiolitis interventions were rolled out and mapped to the Template for Intervention Description and Replication (TIDieR) checklist [25].

### Data collection

Hospital baseline demographics were collected. Quantitative process evaluation data (intervention fidelity) was collected by clinical leads at intervention hospitals (ED and paediatric inpatient units clinical leads) regularly during the implementation period. Qualitative data was collected from clinical leads via an online questionnaire administered on study completion.

Data relating to intervention fidelity, the degree to which interventions were delivered as intended, dose and reach, was collected (minimum monthly) by clinical leads via online entry into a training log (quantitative). Table 2 details the unique fidelity scoring system developed to measure fidelity, with each intervention having the same percentage weighting in the final score. Educational sessions were recorded noting the number of clinicians attending, duration, frequency, who led the session, and modifications made to the educational PowerPoint presentation. Monthly audits (*n* = 7) of the first 20 bronchiolitis presentations (*n* = 10 discharged from ED; *n* = 10 discharged from paediatric inpatient unit) were completed by intervention hospitals. A report was provided with tabulated and graphical compliance by month for their hospital’s total compliance (for all five guideline recommendations), each of the five guideline recommendations, comparisons with previous audits and baseline data, and their hospital benchmarked anonymously to the top performing intervention hospital. Audit and feedback cycle frequency, dissemination methods (written, verbal), frequency of audit report distribution and action planning in light of audit results were recorded. Use of promotional and teaching materials were noted. Intervention hospitals were requested to appoint four clinical leads for the duration of the implementation period (one nursing and one medical clinical lead from ED and paediatric inpatient unit), with guidance provided on suitable clinical lead traits. Clinical leads attended the train-the-trainer day, led delivery of interventions and co-ordinated audit and feedback. Number of clinical leads attending the train-the-trainer day, and whether they remained for the duration of the study were rated. Adherence to completing training logs was assessed at least monthly with regular reminders sent to clinical leads from the research support team.

**Table 2:** Fidelity scoring system for bronchiolitis interventions

Bronchiolitis intervention	Scoring system
1. Clinical lead^a^	Scored out of a maximum of 8 points - 1 point for each clinical lead (maximum 4 points) - 1 point for each clinical lead who maintained engagement with the study for the duration of the intervention year (maximum 4 points)
2. Stakeholder meeting^a^	Scored out of a maximum of 7 points - 1 point for each clinical lead who attended meeting (maximum 4 points) - 1 point for > 90% completion of baseline audit OR 0.5 points for 10–90% completion of baseline audit - 1 point for full explanation of study and study roles provided by research team at stakeholder meeting OR 0.5 points for partial explanation of study and study roles at stakeholder meeting - 1 point for all study leads engaged OR 0.5 points for partial engagement of study leads
3. Train-the-trainer^a^	Scored out of a maximum of 4 points - 1 point for each clinical lead who attended the training day
4. Educational intervention delivery^a^	Scored out of a maximum of 6 points - 1 point for delivery of education to > 80% of medical staff per department - 1 point for delivery of education to > 80% of nursing staff per department OR - 0.5 points for delivery of education to 20–80% of medical staff per department - 0.5 points for delivery of education to 20–80% of nursing staff per department (maximum 4 points) PLUS - 1 point for using provided presentation and key messages per department OR 0.5 points for similar presentation (maximum 2 points)
5. Use of other educational materials^a^	Scored out of a maximum of 10 points - 1 point for using video example of discussing with families a diagnosis of bronchiolitis in education of medical staff per department (maximum 2 points) - 1 point for using video example of discussing with families a diagnosis of bronchiolitis in education of nursing staff per department (maximum 2 points) - 1 point for using fact sheets (CXR, antibiotics, salbutamol) in education of medical staff per department (maximum 2 points) - 1 point for using fact sheets (CXR, antibiotics, salbutamol) in education of nursing staff per department (maximum 2 points) - 1 point for use of promotional materials per department (maximum 2 points)
6. Audit and feedback^a^	Scored out of a maximum of 28 points - 1 point for undertaking each monthly audit (maximum 7 points) - 3.5 points for using written feedback per department for all audits OR 2 points for using written feedback per department for some audits (maximum 7 points) - 3.5 points for using verbal feedback per department for all audits OR 2 points for using verbal feedback per department for some audits (maximum 7 points) - 3.5 points for developing an action plan based on audit data per department for all audits OR 2 points for developing an action plan based on audit data per department for some audits (maximum 7 points)

^a^Equal weighting for each intervention in final total fidelity score e.g. a maximum score for each intervention contributes 16.7% to the final total fidelity score

Qualitative data from the clinical lead questionnaire was collected at study end, gaining feedback from clinical leads on interventions and their delivery. Integrating quantitative and qualitative data with effectiveness findings was undertaken to assist with analysis and interpretation [22]. Additional file 1: Appendix Table 1 describes research questions for each process evaluation domain.

### Data analysis

#### Recruitment and reach

Heterogeneity of clusters (intervention and control hospitals) was assessed quantitatively by comparing hospital type, annual bronchiolitis presentation numbers, staffing numbers and baseline compliance to the five key recommendations from the Australasian Bronchiolitis Guideline. Bronchiolitis intervention reach to clinicians was evaluated through information provided from training logs which clinical leads maintained over the implementation period.

#### Intervention delivery

Data on intervention delivery was analysed descriptively. Individual intervention hospital results are presented as percentage compliance for each intervention and total percentage compliance for all six interventions. A combined hospitals result is presented as percentage compliance for each intervention (mean; standard deviation (SD)) and percentage total compliance for all six interventions (mean; SD). A scoring system was created by the research group to capture fidelity components for the six interventions, with each intervention equally weighted in the total mean fidelity score (Table 2).

### Effect evaluation

We plotted change in primary outcome compliance with all five bronchiolitis guideline recommendations (between 2014/2015 versus 2017 implementation year) for each individual intervention hospital (cluster level) and total mean fidelity score by hospital to assess a possible relationship.

#### Response to intervention

Clinical lead questionnaires were analysed qualitatively using thematic analysis to give insights in to perceptions of the interventions, acceptability, delivery, and receipt. Findings from these questionnaires are reported descriptively using quotations.

## Results

### Recruitment of clusters and cRCT findings

The outcomes of the cRCT are reported in detail elsewhere [19]. In summary, 26 hospitals (Australia = 20; New Zealand = 6) were randomised (intervention group = 13; control group = 13), including 7 tertiary paediatric hospitals (all in Australia). No hospitals withdrew following randomisation. Intervention and control hospitals were well balanced at baseline (Table 3).

**Table 3:** Baseline characteristics from bronchiolitis cluster randomised controlled trial

Characteristics of hospitals	Intervention	Control
**Provider of paediatric care – no. (%)**		
Tertiary	4/13 (31%)	3/13 (23%)
Secondary	9/13 (69%)	10/13 (77%)
**Annual ED presentations per hospital (2017) - Median (IQR)**	61,898 (53,000, 81,635)	69,391 (53,880, 85,413)
**Proportion of ED paediatric presentations per hospital - Median % (IQR)**	25% (20, 31)	21% (20, 24)
**Staffing – Full-time equivalent per hospital (January 2017) - Median (IQR)**		
Medical ED	48 (31, 61)	66 (31, 77)
Nursing ED	84 (72, 105)	116 (75, 132)
Medical paediatric inpatient unit	17 (13, 30)	17 (11, 20)
Nursing paediatric inpatient unit	30 (22, 39)	26 (21, 36)
**Compliance with Australian Bronchiolitis Guideline (pre-intervention) – no. (mean% ± SD)**		
During 2014	790/1238 (64%) (64% ± 15%)	813/1351 (60%) (60% ± 17%)
During 2015	952/1378 (69%) (69% ± 8%)	846/1355 (62%) (62% ± 16%)
During 2016	989/1350 (73%) (73% ± 8%)	874/1331 (66%) (66% ± 14%)

*IQR* Interquartile range, *SD* Standard deviation, *ED* Emergency Department

The primary outcome was compliance with the Australasian Bronchiolitis Guideline during the first 24 h of care (acute care period), with no use of CXRs, salbutamol, antibiotics, glucocorticoids and adrenaline. Implementation year data was collected on 3727 infants. Compliance with the guideline recommendations was 85.1% (95%CI 82.6–89.7%) in intervention hospitals versus 73.0% (95%CI 65.3–78.8%) in control hospitals, with an adjusted risk difference of 14.1% (95% CI 6.5–21.7%, *p* < 0.001) favouring the intervention hospitals.

### Implementation fidelity, dose and reach to clusters and individuals

All 13 intervention hospitals received the six bronchiolitis interventions as per study protocol with total intervention hospital fidelity scores ranging from 55 to 98% (mean 78%; SD 13%) (Table 4).

**Table 4:** Fidelity of bronchiolitis interventions by intervention hospital

Bronchiolitis interventions^a^	Intervention hospitals													
	1	2	3	4	5	6	7	8	9	10	11	12	13	All hospitals (Mean %, SD%)
Clinical leads (%)	100	100	100	100	75	100	100	100	100	100	100	100	100	98, 7
Stakeholder meeting (%)	75	100	100	100	75	75	100	75	100	75	100	100	100	90, 13
Train-the-trainer (%)	75	100	75	75	75	75	100	50	100	25	100	100	100	81, 23
Educational intervention delivery (%)	83	92	42	67	33	58	75	75	83	42	83	100	75	70, 21
Use of other educational materials (%)	90	90	40	80	50	60	60	60	70	40	80	90	40	65, 19
Audit and feedback (%)	68	68	39	68	45	46	46	73	100	46	73	100	73	65, 20
**Mean total fidelity score (%)**	**82**	**92**	**66**	**82**	**59**	**69**	**80**	**72**	**92**	**55**	**89**	**98**	**81**	**78, 13**

^a^A fidelity score for each individual bronchiolitis intervention was calculated, which is represented here as a % of the total possible score. Each intervention has equal weighting in the mean total fidelity score

#### Clinical leads

Intervention hospitals were requested to identify four clinical leads, one nursing and one medical lead in each of ED and paediatric inpatient units for study duration. Twelve (92%) of 13 hospitals achieved this, one hospital had three clinical leads for the duration (fidelity mean 98%; SD 7%). Key clinical lead tasks are detailed in Table 1. Of the 55 clinical leads 42 (76%) responded to the qualitative questionnaire (questionnaire findings are reported descriptively using quotations in italics). Table 5 details characteristics of clinical lead who responded.

**Table 5:** Clinical lead questionnaire response

Clinical leads	***N*** = 55^**a**^	
Response rate by department and clinician group, n (%)	Completed *n* = 42 (76%)	Not completed *n* = 13 (24%)
ED nursing, n (%)	9 (64%)	5 (35%)
ED medical, n (%)	9 (69%)	4 (31%)
Paediatric inpatient unit nursing, n (%)	12 (86%)	2 (14%)
Paediatric inpatient unit medical, n (%)	11 (92%)	2^b^ (8%)
**Response rate by department**		
ED, n (%)	18 (67%)	9 (33%)
Paediatric inpatient, n (%)	23 (89%)	4^b^ (12%)
**Response rate by clinician group**		
Nursing (ED and paediatric inpatient unit), n (%)	21 (75%)	7 (25%)
Medical (ED and paediatric inpatient unit), n (%)	20 (84%)	6^b^ (24%)

*ED* Emergency department

^a^Included all clinical leads throughout study (four clinical leads changed during study due to sickness or left)

^b^One paediatric inpatient unit medical clinical lead never appointed

Clinical leads reported positively on their role, with teamwork in both their department and inter-departmentally viewed as valuable.

*Close working relationships between paediatric and ED members of the study team locally made rolling out a new clinical policy much easier.* (Paediatric inpatient unit, medical).

*I found the use of a clinical lead as "go to" person on the floor was useful and everyone quickly learnt who to go to from the education sessions that we ran with most of the medical and nursing staff.* (ED, nursing).

Time constraints for education, and trying to influence some clinicians’ practise were highlighted as challenges.

*Educating in a busy department is challenging.* (ED, nursing).

*Changing some traditionalists mindset* [what was challenging]*.* (ED, medical).

#### Stakeholder meeting

Research study group members met with clinical leads at each intervention hospital at the beginning of the study to discuss the Australasian Bronchiolitis Guideline, international and local variation in bronchiolitis management, review their hospital’s audit of bronchiolitis compliance (*n* = 40 infants with bronchiolitis), and discuss any anticipated local barriers, with the aim to gain hospital buy-in. Forty-seven (fidelity mean 90%, SD 13%) of the 52 clinical leads attended stakeholder meetings, with a meeting held at each hospital (100%).

#### Train-the-trainer workshop

All clinical leads were requested and funded to attend a workshop facilitated by research group members and credible experts in bronchiolitis and implementation science. Table 1 details workshop content. The bronchiolitis expert role modelled delivery of the educational PowerPoint intervention, emphasising important key-points targeting behaviours and beliefs we were influencing. Forty-two (fidelity mean 81%; SD 23%) clinical leads attended the workshop, with every intervention hospital having at least one clinical lead attend. Feedback on the workshop was overwhelmingly positive.

#### Educational intervention delivery

A PowerPoint presentation with messages addressing key findings from the qualitative study, using behavioural change techniques most likely to effect change was provided. Key messages needing to be conveyed to clinicians were highlighted. Clinical leads aimed to train 80% of clinicians with the PowerPoint presentation within the first month. Five (38.5%) hospitals achieved this, five (38.5%) trained > 50% of staff, and three (23%) trained < 50% staff. Clinical leads mentioned many further informal educational sessions conducted which were not quantified e.g. bedside clinical teaching; at nursing huddles; during one-on-one conversations. All hospitals continued educating clinicians for the duration of the implementation period. Positive feedback on the presentation was reported. Some clinical leads modified the presentation to enable shorter teaching sessions, keeping key message slides. Elements of the presentation felt beneficial were slides detailing evidence supporting the five evidence-based recommendations, and key message slides (with red stickers emphasising importance).

*The most powerful elements were the statistics on each recommendation and why it* [therapies / management processes] *makes no difference.* (Paediatric inpatient unit, nursing).

*The slides with ‘red stickers’* [five key slides detailing why not to use therapies / management processes] *and adverse effects of interventions.* (ED, medical).

#### Use of other educational materials

Additional materials included: a video demonstrating a clinician discussing bronchiolitis with a family; fact sheets (evidence behind no use of CXR, salbutamol and antibiotics); and promotional materials (posters) with use ranging from 40 to 90%. The clinician video received mixed reviews, with some leads finding it useful for junior staff, and others not using it due to time constraints.

*Excellent for medical staff if junior, and not yet developed their own “spiel”.* (ED, medical).

Fact sheets were particularly useful for senior medical clinicians whom clinical leads were struggling to influence. Despite these being designed for health professionals, some clinical leads found them helpful for parents.

*These* [evidence sheets] *were more useful for senior medical staff that questioned the basis for the recommendations.* (ED, medical).

Two posters (detailing guideline recommendations and over-use of therapies) were well utilised. Feedback was positive, with posters displayed in both clinical (for patients/families and clinicians) and non-clinical areas (for staff; displayed on educational boards, in staff tearoom and staff bathroom).

*Posters, particularly the recommendations poster were placed in the doctor write-up area and in the paediatric resus area. Proved to be valuable and effective reminders of what not to do.* (ED, medical).

*They* [posters] *were very well received. They were pretty well visible from every cubicle.* (ED, nursing).

#### Audit and feedback

Monthly audits (*n* = 7) of 20 bronchiolitis presentations were completed by each intervention hospital. Individualised hospital audit reports were produced detailing tabulated and graphical compliance results by month for use of chest x-ray, salbutamol, glucocorticoids, antibiotics and adrenaline, temporal trends, and anonymised benchmarking against the top performing hospital. Dissemination of this report in verbal and written format to clinicians was requested, with action planning and target setting encouraged. All hospitals completed seven audits (100%) with results disseminated back to staff in a variety of methods and frequency. Overall fidelity for audit and feedback ranged from 39 to 100% (fidelity mean 65%; SD 20%) (Table 4).

Clinical leads were positive about the usefulness of audit and feedback, although time to complete audits was challenging. Variation in feedback dissemination strategies was helpful for clinicians, and discussion between departments at handovers or during education sessions was viewed positively.

*Feedback was used to congratulate improvement, but also to suggest re-focus on our treatment after the initial implementation "excitement" faded.* (ED, nursing).

*Feedback was clear, concise, easily understood and distributed by email effectively. Informal discussion regarding individual cases occurred. Results in general were discussed at combined ED and Paediatrics educational sessions.* (ED, medical).

*It was a good reminder about keeping teaching up to date and allowed feedback to staff. It also allowed us to identify misses - a locum paediatric House Officer was not informed of our guidelines.* (Paediatric inpatient unit, medical).

### Effect evaluation

**Fig. 1:**
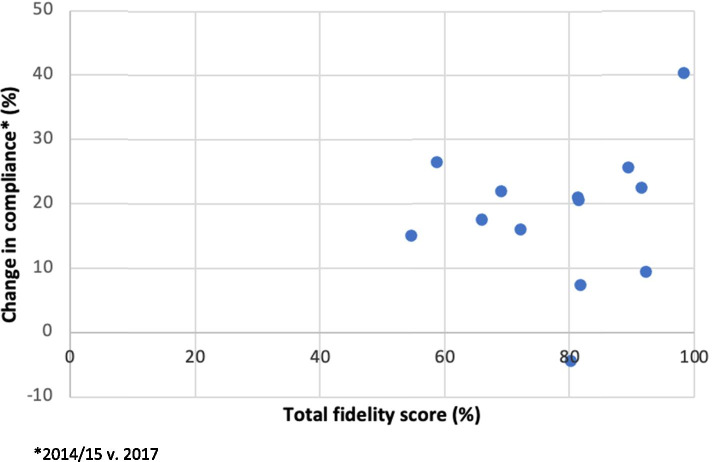
Change in individual intervention hospital bronchiolitis compliance to five guideline recommendations (2014/2015 to 2017) in relation to total intervention fidelity score

All 13 intervention hospitals utilised the six interventions with good adherence to study protocol. This was achieved within clinical lead’s non-clinical time and existing local educational programmes. Figure 1 details bronchiolitis intervention fidelity and change in compliance with all five bronchiolitis guideline recommendations between 2014/2015 to 2017. There is no obvious relationship demonstrated between fidelity (i.e. protocol adherence) and guideline compliance. The vast majority of hospitals (*n* = 12; 92%) improved compliance with bronchiolitis guideline recommendations, with one hospital having reduced compliance despite achieving 80% intervention fidelity. The hospital with the second lowest fidelity score had the second highest compliance improvement, and the hospital with the highest fidelity score had the highest compliance improvement.

Clinical leads were positive regarding the success of the interventions in implementing the five bronchiolitis recommendations:

*Absolutely. There has been a culture shift. It was challenging initially, but gradually changed the mindset.* (ED, medical).

*Implementing the five recommendations was straight forward and successful.* (Paediatric inpatient unit, medical).

*Utilising educators in both departments using the same information meant consistency and increased effectiveness.* (Paediatric inpatient unit, nursing).

Time challenges in educating staff, and maintaining education to cover staff rotations was raised. Medical clinicians were considered harder to influence than nursing, with some nurses being uncomfortable questioning medical decisions:

*Physician dependent. Some physicians excellent at implementing recommendations; some not so.* (ED, nursing).

*Initially widespread acceptance but then old habits came creeping back in through older consultants.* (ED, medical).

*I feel the nurses are getting the information but still not comfortable asking the medical team why they are charting salbutamol.* (Paediatric inpatient unit, nursing)

Minor modifications of the interventions were allowed, with request that key messages remained. Some clinical leads modified the presentation, with 14 (33%) condensing slides to meet time constraints, leaving key messages unchanged.

## Discussion

Process evaluation of de-implementation is rare, yet vital for interventions from successful experimental evaluations to be implemented into real-world clinical practice. This is particularly important where de-implementation is required as this is believed harder than implementation, with calls for more rigorous de-implementation research to be undertaken [12]. Our process evaluation of a cRCT which successfully utilised interventions to de-implement non-evidence-based bronchiolitis management in a real-world clinical setting addresses this deficit. We utilised recommendations from two process evaluation frameworks [20, 23], and a systematic review of process evaluations [22]. This mixed-methods process found that our targeted, theory-informed interventions were delivered with moderate-to-high fidelity (55 to 98%) and were well received by clinical leads. The interventions reached target clinicians and were found to be acceptable. Main challenges were time constraints of delivering interventions within the everyday demands of clinical practice.

The effectiveness of our bronchiolitis interventions has been robustly assessed via a multi-centre cRCT [19]. In this trial of 26 hospitals, with data from 3727 infants, our interventions improved bronchiolitis management by 14.1% (95% CI 6.5–21.7%) in intervention hospitals compared to control hospitals who undertook usual dissemination practices of the Australasian Bronchiolitis Guideline [5]. This absolute change is at the upper end of improvements shown in cRCTs [26]. Our process evaluation, along with our cRCT results confirm our interventions effectiveness in improving treatment of infants with bronchiolitis, and importantly were acceptable to clinicians who rolled it out within existing clinical and non-clinical time, in a real-world clinical environment. These results show promise for widespread use of our intervention where drivers of bronchiolitis management are similar.

Complex interventions, those having multiple interacting components are increasingly used to tackle problems such as evidence-based management of common conditions like bronchiolitis [27]. Process evaluation aims to open the “black box” of implementation studies to tease out why or why not an intervention might work. While they cannot realistically answer all effects of complex interventions, answering key questions well is more valuable than attempting to answer many questions with less certainty [20]. Using a mixed-methods design is encouraged as helps to capture what happened and why, and using theory enables comparisons between studies [22]. Our process evaluation methodology addresses these requests.

Our interventions were implemented and delivered as intended, with 13 intervention hospitals utilising interventions similarly in terms of content, dose and delivery. Importantly, the sequence of intervention delivery was the same in each hospital. Our scoring system ensured accurate and transparent fidelity measurement. The six interventions had equal weighting without assuming one intervention was more effective than another (Table 2). Fidelity use was likely higher than reported, as all intervention use was unlikely to be captured. Data collection timing can be a methodological weakness in process evaluations, with recommendations for data collection pre, during and post the study [22]. A strength of our study was that quantitative fidelity data was collected regularly throughout the study, although some clinical leads required more frequent reminders than a monthly email to complete the training log. Qualitative data was collected immediately after the implementation period, minimising recall bias and reducing challenges of retrospective data collection. Mixed-methods evaluation adds richness to our findings, with interpretation enhanced by the triangulation of qualitative and quantitative data with intervention effectiveness data.

Total fidelity scores ranged between intervention hospitals. All scored > 55% and two thirds scored over 80%, affirming positive reach and receipt of interventions. This represents moderate-to-high fidelity, with others suggesting that 80 to 100% intervention adherence represents ‘high’ fidelity, 51 to 79% represents ‘moderate’ fidelity, and < 50% ‘low’ fidelity [28, 29]. The lack of clear interaction between intervention fidelity and compliance needs cautious interpretation due to the small number of hospitals (Fig. 1). One hospital had a small reduction in overall compliance with an 80% fidelity score, while the hospital with the second highest increase in compliance had the second lowest fidelity score. All hospitals embraced the interventions positively, achieving > 55% fidelity, positive qualitative findings from clinical leads, and a positive cRCT result. Despite no clear interaction between fidelity and improved compliance, we believe a fidelity threshold effect possibly exists. Our positive cRCT results and moderate-to-high fidelity in a real-world setting, suggests that using our interventions with intensity, improvement in compliance with bronchiolitis management can be expected [19].

Intervention hospitals aimed to train 80% of clinicians within the first month, as educating at the end of the bronchiolitis season would have minimal impact. Increased autumn workloads in acute paediatrics and number of staff in large departments, particularly EDs, likely influenced the lower initial education rate. Only five (38%) hospitals achieved the target, although a further five (38%) hospitals achieved > 50% of clinicians trained, and training continued over the duration of the bronchiolitis season. This suggests that a lower education target with continued education is effective. Future recommendations to increase education would include starting bronchiolitis education earlier in the season, mandating education, and utilising online education tools.

All intervention hospitals completed seven audit and feedback cycles, with differences in dissemination of results. The provision of real-time data was viewed favourably to monitor progress and redirect education. Anonymously benchmarking individual hospitals against the top performing hospital added competition, with some hospitals striving to be the top performing hospital. The literature indicates that showing clinicians performance data has improved guideline compliance in other medical conditions [30, 31], with systematic reviews of audit and feedback suggesting small but potentially important improvements (RD 4.3%; IQR 0.5 to 16.0%) [32].

The clinical leads’ role was clearly defined with their importance reinforced during the train-the-trainer day. Clinical lead fidelity was high across all hospitals (mean 98%; SD 6.9%). Clinical leads either alone or in combination with other interventions has shown effectiveness in meta-analysis (*n* = 24 RCTs), with median absolute improvement in care of 10.8% (IQR 3.5 to 14.6%) [33]. Successful leads have been identified as having key attributes: influence, ownership, physical presence, grit, persuasiveness, and participative leadership [34]. Although we did not formally assess clinical lead attributes, we believe many demonstrated these throughout the study.

All clinical leads were invited to complete a questionnaire at study end, giving feedback on the interventions. Similar process evaluation of a complex intervention involving several departments in multiple hospitals, had the majority of questionnaires completed by one clinician on behalf of each hospital, potentially gaining only a single viewpoint [35]. Inviting each of our clinical leads to complete the questionnaire ensured opportunity for all views to be heard. A 76% response rate allowed adequate reflection of views from most clinical leads. Questionnaire findings were overwhelmingly positive regarding interventions. Suggestions for the clinician video and PowerPoint presentation to be shortened are helpful and realistic. Clinical leads were pragmatic by reducing presentation content, ensuring important key messages remained, and using the video judiciously. While we requested hospitals utilise all interventions, we accepted the impact on intervention delivery of busy hospitals over autumn and winter months, and appreciated honesty in describing intervention modifications. Promotional posters had overwhelmingly positive feedback. A systematic review of printed educational materials used alone or compared to no intervention demonstrated small effect, with effectiveness as part of a multifaceted intervention being uncertain [36]. Our positive feedback and positive cRCT result suggest that posters, being easy and low-cost to produce, be considered as part of future intervention packages.

A strength of our study is that no hospitals withdrew post randomisation, suggesting clinicians’ and hospitals’ commitment to reducing low-value care when treating infants with bronchiolitis, and that interventions were appropriate and realistic for the real-world setting of de-implementation in acute paediatrics. We also acknowledge study limitations. Clinical lead feedback was positive, but there may be response bias as perspectives of the leads who did not respond is unknown. We did not obtain feedback from clinicians who received study interventions from clinical leads. However, all clinical leads were also practicing clinicians, and we surmised their feedback would be similar to their clinician colleagues.

Attempting to identify one intervention as superior to the others is tempting, but not possible as the six interventions were delivered as a package and not independent. However, opening the ‘black box’ on our de-implementation study has given insight into what worked, why, and potential barriers to implementation. Findings are important as we scale-up dissemination of our interventions to improve the treatment of infants with bronchiolitis, with modifications addressing time challenges of education delivery. A co-ordinated approach, utilising national and international networks to disseminate our findings and interventions being required to optimise translation [37].

## Conclusion

This process evaluation found our bronchiolitis interventions were delivered within the ED and paediatric context as intended, received positively, with good reach. All six interventions were undertaken, resulting in improvement in the treatment of infants with bronchiolitis. Time constraints for intervention delivery posed challenges. However, clinical leads were adaptable to ensure key intervention components were delivered. These results provide guidance to researchers and clinicians in utilising our interventions in ED and paediatric inpatient settings and have wider implications for de-implementation science in general.

## Supplementary Information

**Additional file 1: Appendix Table 1** Components and methods of process evaluation. **Appendix Table 2** Bronchiolitis intervention detail based on Template for Intervention Description and Replication (TIDieR). **Appendix Table 3** Clinical lead questionnaire.

## Data Availability

Not applicable.
