# Supplementary material for: Process evaluation of a cluster randomised controlled trial to improve bronchiolitis management – a PREDICT mixed-methods study
Source: BMC Health Serv Res. 2021 Nov 29;21:1282. doi: 10.1186/s12913-021-07279-2 (PMC8628472; doi:10.1186/s12913-021-07279-2)
Supplement: Supplementary file 1 — Additional file 1: Appendix Table 1 Components and methods of process evaluation. Appendix Table 2 Bronchiolitis intervention detail based on Template for Intervention Description and Replication (TIDieR). Appendix Table 3 Clinical lead questionnaire. [file 12913_2021_7279_MOESM1_ESM.docx]

**Appendix Table 1** Components and methods of process evaluation

| **Evaluation domain** | **Research questions** | **Research methods** | **Data collection tools** | **Data collection timing** |
| --- | --- | --- | --- | --- |
| Recruitment of clusters^1^ | How are clusters sampled and recruited?  Who agrees to participate?  Why do clusters agree to participate (or not)? | Descriptive analysis of recruited clusters.  Baseline data. | Hospital characteristics and baseline data collected (intervention and control) | Pre-intervention. |
| Recruitment and reach in individuals^1,2^ | Who actually receives the intervention in each setting? Are they representative?  Why do clusters achieve the pattern of reach they do? Do they introduce selection bias? | Descriptive analysis of intervention delivery via training log and fidelity scores. | Clinical leads at intervention hospitals regularly recorded interventions utilised throughout the implementation period (at least monthly), frequency, delivered to which clinicians, duration. | During and post-intervention. |
| Delivery to clusters^1^ | What intervention is actually delivered to clusters? Is it the one intended by researchers? | Descriptive analysis of intervention delivery via training log and fidelity scores. | Training log data was entered in to fidelity assessment tool at the end of the study to give a fidelity score for each intervention, and overall fidelity percentage for intervention package. | During and post-intervention. |
| Delivery to individuals^1,2^ | What intervention is actually delivered to individuals? Is it the one intended by researchers? | Descriptive analysis of intervention delivery via training log and fidelity scores. | Clinical leads at intervention hospitals regularly recorded interventions utilised throughout the implementation period (at least monthly), frequency, delivered to which clinicians, duration. | During and post-intervention. |
| Response of clusters^1^ | How is the intervention adopted by clusters? | Qualitative analysis of clinical lead questionnaires. | A questionnaire sent to all clinical leads at the end of the study. | Post-intervention. |
| Response of individuals^1^ | How does the target population respond? | As above.  cRCT results assessing compliance with five guideline recommendations. | As above.  cRCT data collection on infants with bronchiolitis. | Post-intervention. |
| Maintenance^1^ | How and why are these processes sustained over time (or not)? | Qualitative analysis of clinical lead questionnaires. | See above re intervention data being regularly collected over study period. | During and post-intervention. |
| Unintended consequences^1^ | Are there unintended changes in processes and outcomes, both related to the trial intervention and unrelated care? | Descriptive analysis of training log and clinical lead questionnaire.  cRCT results. | Information from training log and clinical lead questionnaire.  cRCT results. | During and post-intervention. |
| Context^1,2^ | What is the wider context in which the trial is being conducted? | Consideration that trial being conducted in real-world acute care setting during busy bronchiolitis season. | Data from clinical leads pre and during trial. | Pre and during intervention. |
| Theory^1,2^ (Causal Mechanisms) | What theory has been used to develop the intervention? Can a theory be considered to interpret the effects of the intervention? | Theoretical Domains Framework (TDF) used to guide and analyse clinician interviews aiming to identify barriers and facilitators of bronchiolitis management. Behaviour change techniques linked to TDF domains, used to guide intervention development.  Systematic reviews of interventions used to guide intervention development. | Clinician interviews. | Pre-intervention |
| Effectiveness^1,2^ | What are the effects on primary and secondary outcomes? | cRCT results. | Implementation period data collected on 3,727 infants with bronchiolitis to evaluate effectiveness of interventions in improving compliance with bronchiolitis guideline. | Post-intervention. |

cRCT = cluster randomised controlled trial

TDF = Theoretical Domains Framework

^1^Adapted from Grant *et al*^23^

^2^Adapted from Medical Research Council guideline for evaluating complex interventions ^20^

**Appendix Table 2** Bronchiolitis intervention detail based on Template for Intervention Description and Replication (TIDieR)

1. **Name**

Bronchiolitis interventions.

1. **Why**

To improve the evidence-based treatment of infants with bronchiolitis by reducing the unnecessary use of chest x-ray (CXR), salbutamol, glucocorticoids, antibiotics, and adrenaline.

1. **What**

**Educational intervention material**

- **Australasian Bronchiolitis Guideline** in electronic and hard copy format (in full and bedside clinical versions). Rationale: Improve knowledge; change beliefs; improve confidence.
- **PowerPoint presentation covering:**
  - Overview of bronchiolitis.
  - The Australasian Bronchiolitis Guideline and key supporting evidence.
  - Data demonstrating international and local variation in bronchiolitis management.
  - Each of the five evidence-based recommendations from the Australasian Bronchiolitis Guideline with key messages to be delivered to clinicians (presentation utilised behaviour change techniques most likely to effect change, having been identified from qualitative clinician interviews analysed using the Theoretical Domains Framework (TDF)).
  - Supplementary PowerPoint slides providing additional evidence supporting guideline recommendations.

Rationale: Improve knowledge; increase skills; change beliefs; feedback on performance; address barriers and enablers to evidence-based management; reinforce importance of evidence-based management and consequences of not following recommendations; positive reinforcement.

**Additional educational materials**

- **Clinician training video:**
  - Role modelling how to discuss bronchiolitis and its management with family.

Rationale: Demonstrate/role model clinician behaviour; increase skill; provide motivation.

- **Evidence fact sheets:**
  - Providing detailed evidence supporting no use of CXR, salbutamol and antibiotics.

Rationale: Improve knowledge; change beliefs.

- **Promotional materials:**
  - Poster promoting the Australasian Bronchiolitis Guideline and five key guideline recommendations.
  - Poster presenting data illustrating variation in evidence-based treatment of infants with bronchiolitis.

Rationale: Reminder/prompt of recommended management; feedback on performance; provide motivation.

- **Parent/caregiver bronchiolitis information sheet:**
  - Providing information on bronchiolitis, expected illness trajectory, supportive care, and guidance for when to seek medical review.

Rationale: Improve knowledge; increase skill and confidence; provide encouragement and support.

1. **What (procedure)**

- **Clinician leads:**
  - Four clinical leads, one nursing and one medical lead from each of the emergency department (ED) and paediatric inpatient teams for the duration of implementation period.
  - Key tasks included:
    - Attend train-the-trainer one day workshop.
    - Lead delivery of educational intervention and additional educational materials to clinicians.
    - Oversee completion of monthly audit and feedback.
    - Co-ordinate interventions over implementation period.

Rationale: Provide consistent credible, influential, and trustworthy leadership; increase knowledge and skills through education, influence and persuasion; clinical leads ensured interdisciplinary and interdepartmental coverage.

- **Stakeholder meeting:**
  - Aimed to gain site buy-in at an organisational, senior leadership and senior clinician level.
  - Study team met with four clinical leads, and key medical and nursing stakeholders at each site at the beginning of the study to discuss:
    - The Australasian Bronchiolitis Guideline
    - Data demonstrating international and local variation in bronchiolitis management.
    - Review and discussed the site’s audit of compliance with five key bronchiolitis recommendations.
    - Discuss any anticipated local barriers to the interventions.

Rationale: Create site buy-in; provide feedback on current management; knowledge of own practice variation is likely to drive change; increase knowledge of intervention process; identify and address any potential barriers.

- **Train-the-trainer workshop:**
  - One day workshop for all four clinical leads (one nursing and one medical lead from each of the ED and paediatric inpatient team).
  - Workshop content included:
    - The Australasian Bronchiolitis Guideline and key supporting evidence.
    - Implementation science and implementation research.
    - Findings from qualitative study identifying factors influencing bronchiolitis management.
    - Rationale for interventions (detailed stepped process using TDF, mapping to behaviour change techniques, and informed by systematic reviews of intervention components).
    - Role modelling of how interventions were to be delivered.
    - Discussing the responsibilities and importance of clinical lead.
    - Planning time for clinical leads from ED and paediatric inpatient area to work together (encouraging interdisciplinary and interdepartmental communication and relationships).

Rationale: Improve knowledge; change beliefs; optimise professional interdisciplinary and interdepartmental relationships; motivate clinical leads as drivers of change.

- **Audit and feedback:**
  - Sites completed seven monthly audit cycles of 20 infants who presented during the previous month (the first 10 discharged from ED; the first 10 discharged from paediatric inpatient unit) for each month of the implementation period.
  - Each site received a monthly audit report providing tables and graphs detailing:
    - Their site’s compliance in the treatment of infants with bronchiolitis for:
      1. Number of presentations which complied with all five guideline recommendations.
      2. Number of presentations which complied with each of the five individual guideline recommendations.
      3. Comparison of site’s current and previous month’s audit, and baseline audit (from stakeholder meeting) for 1 and 2.
      4. Their site benchmarked against the top performing site for 1, 2 and 3.
  - Audit reports disseminated by clinical leads in ED and paediatric inpatients to their staff in verbal and written format.
  - Clinical leads encouraged to use audits to action plan and set targets for improvement.

Rationale: Provide real-time feedback on targeted behaviours; motivate by benchmarking; promote goal/target specific action planning to optimise on-going improvement; increase knowledge; change beliefs.

1. **Who provided**

- Train-the-trainer workshop facilitated and delivered by research team members (included senior nurses, doctors, and implementation researcher) and clinical opinion leaders (experts in bronchiolitis management).
- Interventions delivered at sites by four clinical leads (medical and nursing, from ED and paediatric inpatient team) who had undergone training at the train-the-trainer workshop, or delivery overseen by clinical leads as appropriate.
- Clinical leads were selected by ED and paediatric inpatient units – ‘ideal characteristics’ of a clinical lead were discussed with sites.

1. **How**

- Australasian Bronchiolitis Guideline in electronic and hard copy format (in full and bedside clinical versions) provided to ED and paediatric inpatient team to utilise and disseminate within their teams.
- PowerPoint presentation delivered face-to-face to all nursing and medical staff within ED and paediatric inpatient teams.
- Clinician video shown to nursing and medical staff within ED and paediatric inpatient teams.
- Promotional materials displayed in ED and paediatric inpatient units to prompt memory of the guideline recommendations and highlight variation in bronchiolitis management.
- Audit results and feedback provided to nursing and medical staff within ED and paediatric inpatient teams in verbal and written form: one-one-one, in small or larger group meetings, via email, in departmental bulletins or quality updates. Action planning and target setting for improvement encouraged.
- Parent/caregiver bronchiolitis information sheet available for sites to use in ED and paediatric inpatient units.

1. **Where**

- All interventions delivered and utilised in ED and paediatric inpatient units.
- PowerPoint presentation delivered face-to-face to all nursing and medical staff within ED and paediatric inpatient teams during nursing and medical meetings, educational sessions, grand rounds, departmental daily huddles.

1. **When and how much**

- Implementation period was from May to November 2017 and included:
  - PowerPoint presentation (10-30 minutes duration) – clinical leads aimed to educate 80% of nursing and medical staff within their ED and paediatric inpatient teams in the first month, then on-going education to ensure all nursing and medical staff educated over the duration of the implementation period.
  - Additional educational materials utilised in ED and paediatric inpatient units over the duration of the implementation period.
  - Audit and feedback – seven monthly audit reports completed with results disseminated by clinical leads to nursing and medical staff within ED and paediatric inpatient teams.

1. **Modifying**

- Educational intervention presentations were streamlined to meet local site scheduling constraints.
- Slight variation in materials was allowed, ensuring that five guideline recommendations and their key messages remained and were reinforced.
- Sites contextualised promotional material for their ED and paediatric inpatient units.
- Parent/caregiver bronchiolitis information sheet could have site logo and contact details added.

1. **Modification**

- No modifications were made by the research team during the implementation period.

1. **How well (planned)**

- Clinical leads from ED and inpatient paediatric teams maintained a log during the implementation period detailing:
  - Educational sessions completed, number and type of clinicians, which department, duration and materials utilised.
  - Audit and feedback dissemination – frequency, mode of dissemination, number and type of clinicians.
  - Promotional materials used and duration of use.

1. **How well (actual)**

- All sites had clinical leads attend the train-the-trainer workshop and for the duration of the implementation period.
- All sites delivered the interventions as per study protocol.
- 5/13 sites educated 80% of nursing and medical staff in the first month; all sites continued education over the duration of the implementation period.
- All sites completed all seven audits and disseminated to nursing and medical staff.

**Appendix Table 3** Clinical lead questionnaire

1. Did you attend the train-the-trainer day for the study?
2. Can you tell us whether you felt the project was successful at your site?

- i.e. implementing the 5 key recommendations of the bronchiolitis guideline into your department?

1. How useful did you find the 'Australasian bronchiolitis bedside clinical guideline'?

- Where did you keep it? Was it accessible in hard copy or electronic copy to staff?

1. How useful did you find the power point training presentation slides provided?

- Was this used in your department? Which elements worked well? Which elements did not work so well?

1. How useful did you find the 'Discussion with families' video?

- How did your staff group/s respond to it? How did you find the length?

1. How useful did you find the three fact sheets? (salbutamol and steroids; chest x-rays and antibiotics; parent information sheets)

- Were these used outside of the training sessions? Were these available to staff in your department somewhere? How did parents respond to the parent information?

1. Did you use the posters (guideline poster; recommendations poster)?

- Did staff / patients comment on them? Where did you put them?

1. How useful did you find the audit and feedback process?

- How were the reports reviewed by staff in your department? Did they appear to understand the reports? Were the results discussed? Were individual cases that were non-compliant reviewed / discussed? Was there cross departmental (i.e. between emergency department and paediatric inpatients) discussion about the results?

1. Were there technical / information technology issues in obtaining the records needed for Audit and Feedback?

- If so, please describe

1. In your role as a clinical lead in your department, your role was to provide training and to lead the study on the floor.

- What do you think worked well? What didn't work well? What did you find most challenging? Were you adequately supported by others at your site?

1. Did you meet up with the other site clinical leads at your site after the training day?

- If so, how often did you meet? What was covered during these meetings? Were there other people outside the clinical leads group who became involved in study activities? / study discussions? E.g. senior leadership staff, data entry staff etc. If so, how many / medical or nursing?

1. Did you have contact with ANY other sites (intervention or control) in the study outside of your hospital?

- If so, was this helpful? What did it entail?

1. Please feel free to provide other comments regarding your personal or your site's involvement in this study.
